# Self‐Assembling Cationic Lipopeptides for the Construction of Functional Vesicular Gene‐Delivery Systems

**DOI:** 10.1002/smll.74067

**Published:** 2026-06-05

**Authors:** Federica A. Souto‐Trinei, Alba Ramil‐Bouzas, Paco Fernández‐Trillo, Ana Rey‐Rico, Roberto J. Brea

**Affiliations:** ^1^ Bioinspired Nanochemistry (BioNanoChem) Group CICA – Centro Interdisciplinar de Química e Bioloxía Departamento de Química Facultad de Ciencias Universidade da Coruña A Coruña Spain; ^2^ Gene and Cell Therapy (G‐CEL) Research Group CICA – Centro Interdisciplinar de Química e Bioloxía Departamento de Biología Facultad de Ciencias Universidade da Coruña A Coruña Spain

**Keywords:** gene delivery, lipopeptide, self‐assembly, synthetic cell, vesicle

## Abstract

Delivering nucleic acids across cellular membranes remains a central challenge. Although synthetic gene carriers that mimic living cells are gaining interest, creating robust and biocompatible compartments continues to be technically demanding and limited in scope. Here, we present a new class of cationic lipopeptides that self‐assemble into stable, functional membrane structures, offering a versatile platform for gene‐delivery applications. These lipopeptides combine the biological activity and programmability of peptides with the self‐organizing behavior of lipids, yielding functional vesicular structures that emulate cellular compartments and act as powerful non‐viral vectors. We demonstrate that these assemblies efficiently sequester and deliver nucleic acids, achieving high transfection efficiency with minimal cytotoxicity in HEK 293T cells, and importantly, extending this performance to more challenging cellular models such as mesenchymal stem cells (MSCs). Our findings highlight the potential of self‐assembling cationic lipopeptides as modular building blocks for generating robust, biocompatible, and programmable synthetic cells capable of delivering diverse nucleic acids.

## Graphical abstract

Cationic lipopeptides self‐assemble into stable, cell‐like vesicles that merge peptide programmability with lipid self‐organization. These biocompatible compartments efficiently sequester and deliver nucleic acids, enabling high transfection efficiency with low‐toxicity in both HEK293T cells and mesenchymal stem cells (MSCs). This approach provides a modular, non‐viral platform for synthetic cells and gene delivery.

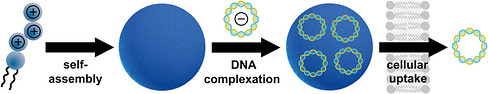

## 1 Introduction

The delivery of nucleic acids across cellular membranes is a central challenge in contemporary science and medicine [1]. Introducing genetic material into cells has transformed modern research and represents a breakthrough with far‐reaching implications for both basic and applied studies [2]. Although viral vectors provide high transduction efficiency and long‐term gene expression in vivo [3], their use is limited by several drawbacks, including strong immunogenicity, toxicity, risks of insertional mutagenesis, and restricted packaging capacity [4].

Non‐viral vectors have emerged as safer alternatives, overcoming several limitations of viral systems [5]. Traditional cationic lipid structures mimic the amphiphilic balance of natural gene carriers, effectively condense nucleic acids through electrostatic interactions, and integrate well with cellular membranes [6]. However, optimizing the number, type and location of cationic lipid residues remains challenging, with only a limited number identified as effective to date [7, 8, 9, 10]. Accordingly, alternative non‐viral systems – including cationic polymer assemblies [11, 12, 13, 14], niosomes [15, 16], and nanoparticles [1, 17] – have been developed. Although these platforms improve stability and tunability [7], they often lack essential features such as specific biological signaling and biodegradability, limiting their applicability [5]. Consequently, there is an urgent need for novel non‐viral vectors capable of targeting hard‐to‐transfect cells while enhancing transfection efficiency and minimizing toxicity [18, 19]. Despite increasing interest in synthetic carriers that emulate the structure and encapsulation/release performance of living cells [20, 21, 22, 23], simple and versatile strategies to create such delivery compartments remain both challenging and limited in scope [24].

**FIGURE 1:**
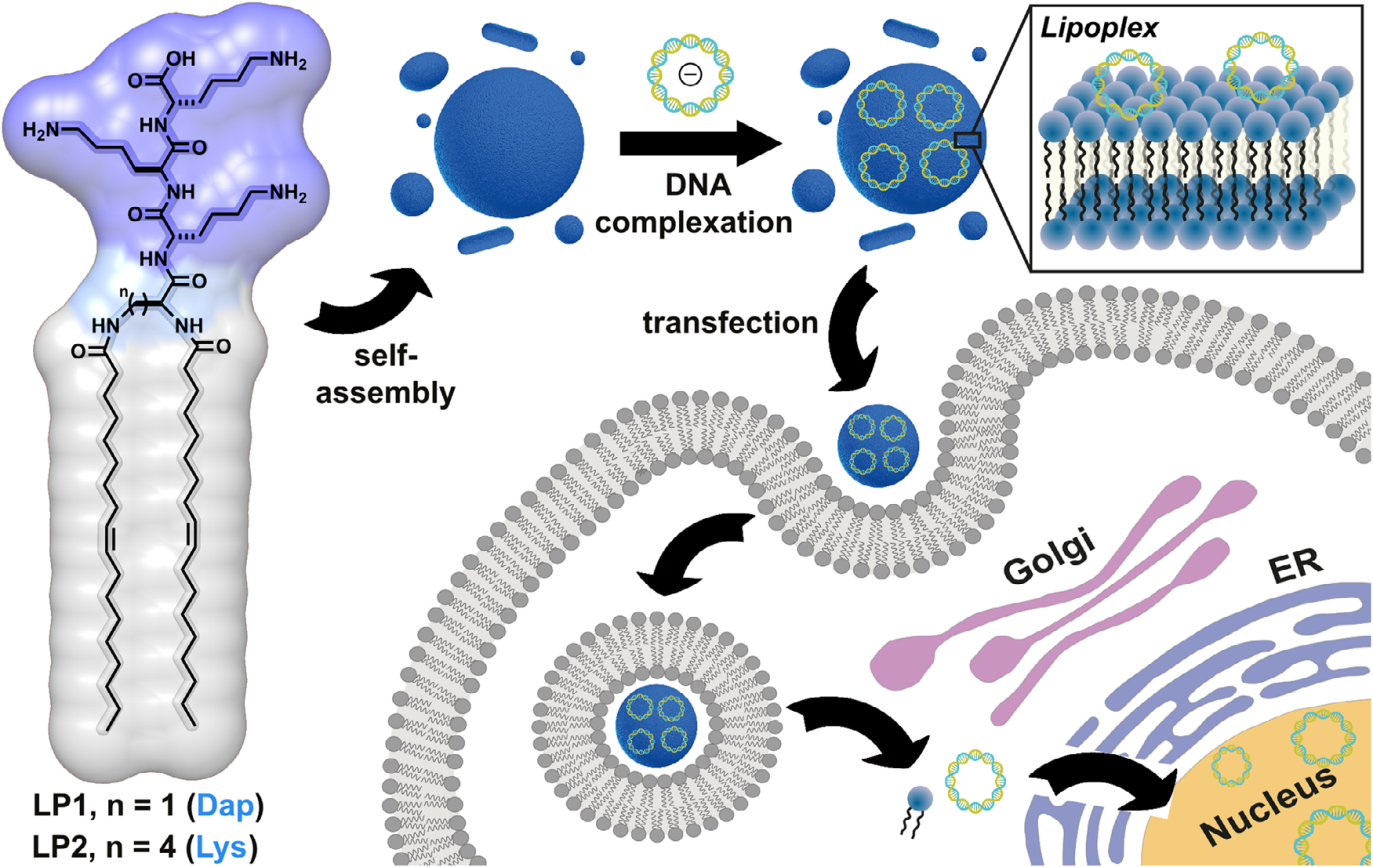
Self‐assembling cationic lipopeptide vesicles as non‐viral gene delivery vectors. Upon sequestering negatively charged nucleic acids, lipopeptide (**LP1** or **LP2**) nanocarriers are internalized, and the genetic cargo is released into the cytoplasm, ultimately reaching the nucleus.

Self‐assembling cationic lipopeptides offer valuable building blocks for developing safer and more effective gene‐delivery carriers [25, 26, 27]. By combining the biological activity and programmability of peptides with the self‐organizing properties of lipids, these amphiphiles can spontaneously form functional cell‐like structures capable of efficiently sequester and delivering nucleic acids. Building on this concept, we present here a new class of cationic lipotetrapeptides that self‐assemble into stable, functional membrane structures, providing a versatile platform for membrane‐based gene‐delivery systems (Figure 1). These lipopeptides integrate peptide functionality and tunability with lipid structural organization to generate highly functionalized vesicular structures.

## 2 Results and Discussion

### 2.1 Synthesis of Lipopeptides

Recent studies have explored phospholipid‐like molecules with unconventional head groups [28, 29, 30], enabling new applications in biomedicine, sensing, and nanotechnology [31, 32, 33]. Among these, amino acid‐ and peptide‐lipid conjugates that spontaneously self‐assemble into robust, micron‐sized vesicular structures are of particular interest due to their functional tunability and stability across a broad range of pH and temperatures [34, 35]. Taking this into consideration, we first synthesized two lipotetrapeptides (**LP1** and **LP2**) following standard solid‐phase peptide protocols (Scheme S1) [35, 36]. The compounds were fully characterized by high‐performance liquid chromatography (HPLC) (Figure S1), mass spectrometry (MS) (Figures S2 and S3), Fourier transform infrared spectroscopy (FTIR) (Figure S4), and circular dichroism (CD) (Figure S5). These lipopeptides differ only in the diacylated residue – L‐2,3‐diaminopropionic acid (L‐Dap) for **LP1** vs. L‐Lysine for **LP2** (Figure 1) –, which structurally organizes the hydrophobic tails. This minor difference of three methylene units enables investigation of the influence of the cationic core and spacer on membrane packing and stability.

### 2.2 Characterization of the Lipopeptide Vesicular Structures

**FIGURE 2:**
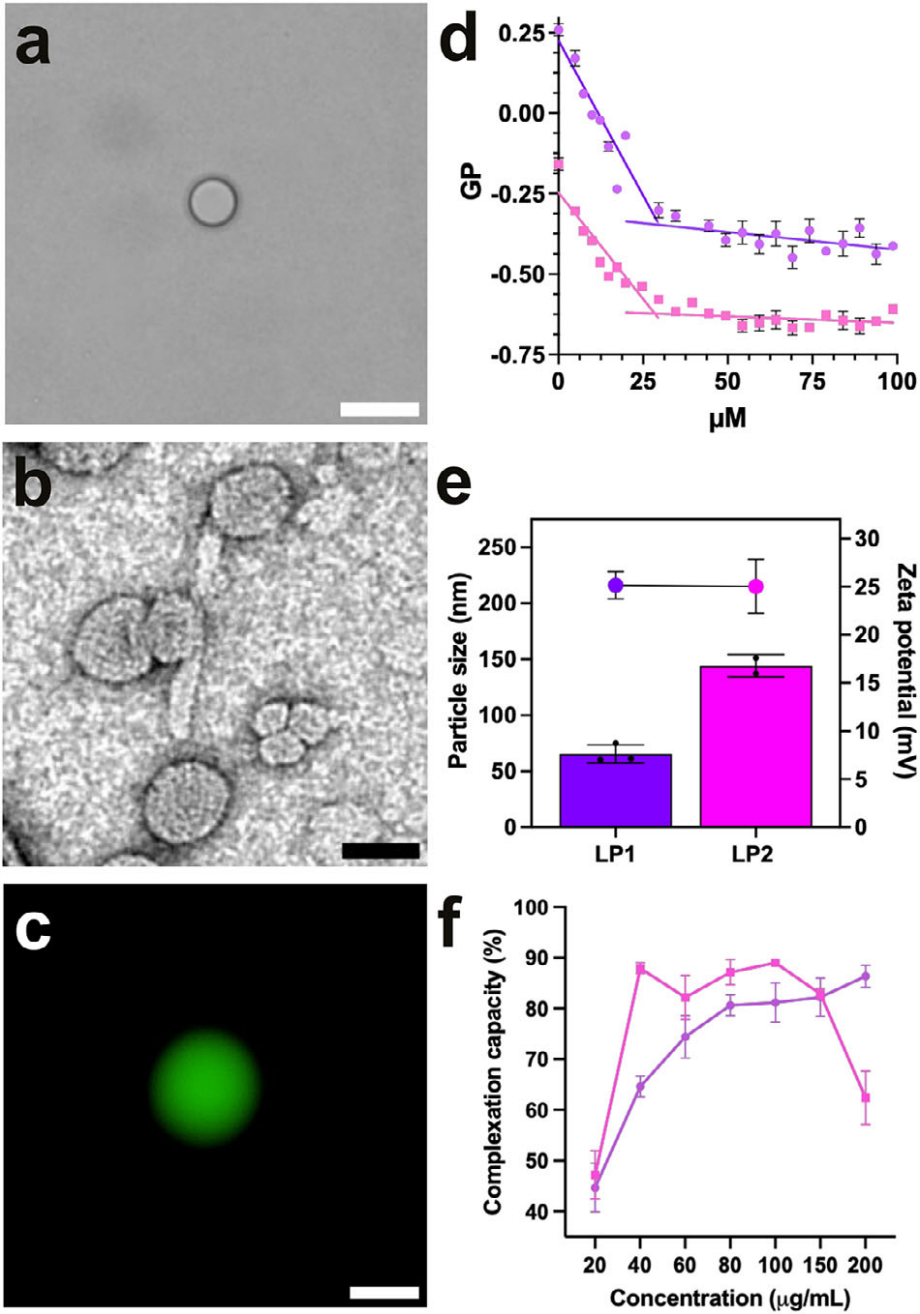
Characterization of cationic lipopeptide vesicular structures. (a) Phase‐contrast image of **LP1** vesicles. Scale bar denotes 5 µm. (b) Transmission electron microscopy (TEM) image of negatively stained **LP1** vesicles. Scale bar denotes 100 nm. (c) Fluorescence microscopy image demonstrating encapsulation of HPTS in **LP1** vesicles. Scale bar denotes 5 µm. (d) Critical aggregation concentrations (CACs) of **LP1** (*purple*) and **LP2** (*magenta*). (e) Particle size distribution determined by DLS *(left)* and zeta potential measured by ELS *(right)* for **LP1** (*purple*) and **LP2** (*magenta*) vesicular nanoparticles produced using the microfluidic‐based platform TAMARA. (f) p*lacZ* complexation efficiency of **LP1** (*purple*) and **LP2** (*magenta*).

We next explored the self‐assembly properties of **LP1** and **LP2** through a comprehensive analysis of the morphology, size, and surface charge of the resulting supramolecular structures. Hydrated samples of the purified compounds were first analyzed using phase‐contrast microscopy, which revealed the spontaneous formation of well‐defined membrane‐bound assemblies (Figure 2a, Figure S6). Vesicle formation was consistently observed for both lipopeptides, indicating that structural differences in the amino acid side chains did not hinder their ability to self‐organize into micron‐sized vesicles in aqueous media. Negative‐staining transmission electron microscopy (TEM) further corroborated the formation of vesicular structures (Figure 2b, Figure S7). Encapsulation efficiency was evaluated using 8‐hydroxypyrene‐1,3,6‐trisulphonic acid (HPTS) as a fluorescent hydrophilic probe. Fluorescence microscopy confirmed successful dye loading within the corresponding lipopeptide vesicles (Figure 2c, Figure S8).

The critical aggregation concentrations (CACs) of **LP1** and **LP2** were determined to be 29.6 and 28.4 µM, respectively, using a method based on the solvatochromic fluorescent dye Laurdan (Figure 2d) [30]. Although **LP1** exhibits a slightly higher CAC than **LP2**, DLS analysis (Figure 2e) revealed a substantial difference in nanoparticle size (69 nm for **LP1** vs. 188 nm for **LP2**), likely resulting from distinct membrane packing caused by the substitution of Dap for Lys (Figure 1) [37]. Both systems, however, displayed the same Z‐potential (25.16 mV), with this positive surface charge conferred by the three consecutive lysine residues at the C‐terminus of the peptide sequences.

### 2.3 Stability of **LP1** and **LP2**

The stability of both lipopeptides under biologically relevant conditions was evaluated by incubation in fetal bovine serum (FBS) (Figure S9). Briefly, each lipid film was hydrated at 37 °C, and aliquots were collected at different time points. The samples were then analyzed by HPLC, monitoring absorbance at 210 nm.

Although slight changes in the chromatographic profiles were detected over time, these were minor and did not significantly affect the proportion of intact compound. Overall, the lipopeptides remained largely stable under FBS conditions.

### 2.4 Transfection of Nucleic Acids Mediated by Lipopeptide Vesicles

#### 2.4.1 Lipopeptide/pDNA Complexes Formation

Considering the identical surface charge of both lipopeptide vesicles, we anticipated minimal variation in their DNA complexation capacity. However, complexation assays with p*lac*Z (7,8 kb) revealed a clear difference in their concentration‐dependent performance (Figure 2f, Figure S10). **LP2** achieved maximum complexation (∼90%) at lower concentrations (20 µg/mL) and maintained high efficiency across a broad range. In contrast, **LP1** displayed a slightly lower complexation capacity (∼85%), although this was still sufficient for subsequent transfection experiments. It is worth noting that the hydration method was selected as the model strategy for complex formation due to its well‐established and straightforward protocol.

#### 2.4.2 HEK 293T Cells

##### 2.4.2.1 Cytotoxicity and Transfection Efficiency of Lipopeptide/placZ Complexes

The efficiency of the lipopeptide vesicles as non‐viral gene delivery vectors was evaluated using the well‐established HEK 293T cell line. In vitro transfection assays were first conducted with the reporter gene p*lac*Z, which encodes β‐galactosidase (β‐Gal) (Figure 2). Intracellular delivery of this enzyme is particularly challenging due to its limited ability to form stable complexes with conventional nanocarriers, a consequence of its large molecular size (∼430 kDa) [38]. Therefore, developing an effective strategy to deliver p*lac*Z and achieve β‐Gal expression within cells would be highly valuable.

**FIGURE 3:**
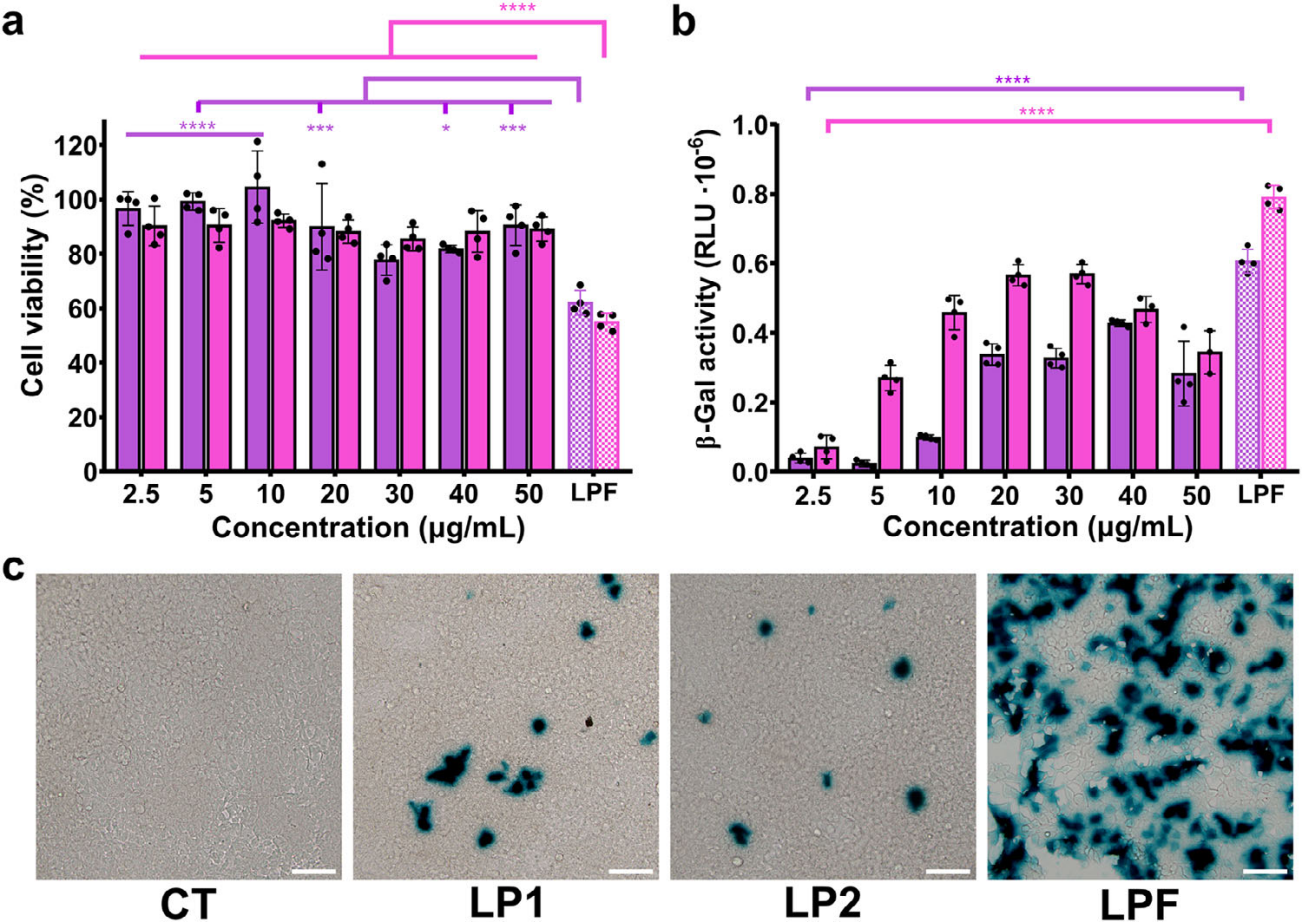
Transfection of p*lacZ* into HEK 293T mediated by cationic lipopeptide‐based synthetic cells. (a) Cell viability of lipopeptide [**LP1** (*purple*) or **LP2** (*magenta*)]/pDNA (p*lacZ*) complexes. (b) β‐galactosidase (β‐Gal) activity of **LP** **1** *(purple)* and **LP** **2** *(magenta)* lipopeptide/p*lacZ* complexes formed at different concentrations. (c) Microscopy images of β‐Gal staining (magnification: 20X; scale bar: 200 µm) showing successful *lac*Z transfection in HEK 293T cells after 3 h of incubation for both **LP1** and **LP2**/pDNA complexes. The commercial reagent Lipofectamine (LPF) served as a positive transfection control. As expected, cells transfected in the absence of lipopeptides or LPF showed no β‐Gal expression (negative control CT). Values (*n* = 4) are presented as mean ± standard deviation (SD). * Depicts p<0.05, **p < 0.01, ***p < 0.001, and ****p < 0.0001 compared with the indicated LPF group.

The cytotoxicity and transfection efficiency of **LP1** and **LP2** were evaluated across the same concentration range (Figure 3a,b, respectively). The highest transfection level for the **LP1**/p*lacZ* complexes was observed at 40 µg/mL (42796.750 ± 901.609), although this did not reach the positive control [Lipofectamine (LPF): 60590.000 ± 3434.086; *p* < 0.0001]. Notably, all tested concentrations maintained cell viability percents above 75%, exceeding that of the positive control (62.090 ± 4.560%). Therefore, this combination of low cytotoxicity and high transfection capability positions **LP1** vesicles as a safe and effective non‐viral gene delivery candidate. Alternatively, the highest transfection levels for the **LP2**/p*lacZ* complexes were observed at 20 and 30 µg/mL (56542.000 ± 3054.690 and 56900.500 ± 2789.527, respectively), where the efficiency plateaued. Similar to **LP1**, the **LP2**/p*lacZ* complexes did not surpass the positive control (LPF: 79061.000 ± 3380.640; *p* < 0.0001). Cytotoxicity followed a similar trend, with all tested concentrations maintaining cell viability values above 75%, while the positive control exhibited the lowest ones (55.041 ± 3.060%; *p* < 0.0001). These findings suggest that the spatial separation of the hydrophobic tails and the resulting differences in membrane packing contribute to the distinct transfection efficiencies observed between **LP1** and **LP2** vesicular assemblies. Such structural variations appear to modulate how each lipopeptide interacts with and condenses genetic material, ultimately influencing their capacity to mediate effective cellular delivery.

##### 2.4.2.2 Cytochemical Detection of β‐Galactosidase Activity

Cytochemical analyses performed using a β‐Gal staining kit corroborated the suitability of the lipopeptide vesicles as effective gene carriers. In situ transfection efficiency was evaluated under an inverted microscope through direct colorimetric observation, where higher β‐Gal expression corresponded to more intense staining, indicating greater transfection capacity (Figure 3c). The optimal concentrations of **LP1** (40 µg/mL) and **LP2** (20 µg/mL) were examined using cells transfected with LPF and untransfected cells as a positive and negative controls, respectively.

##### 2.4.2.3 Internalization Mechanism of Lipopeptide/placZ Complexes

**FIGURE 4:**
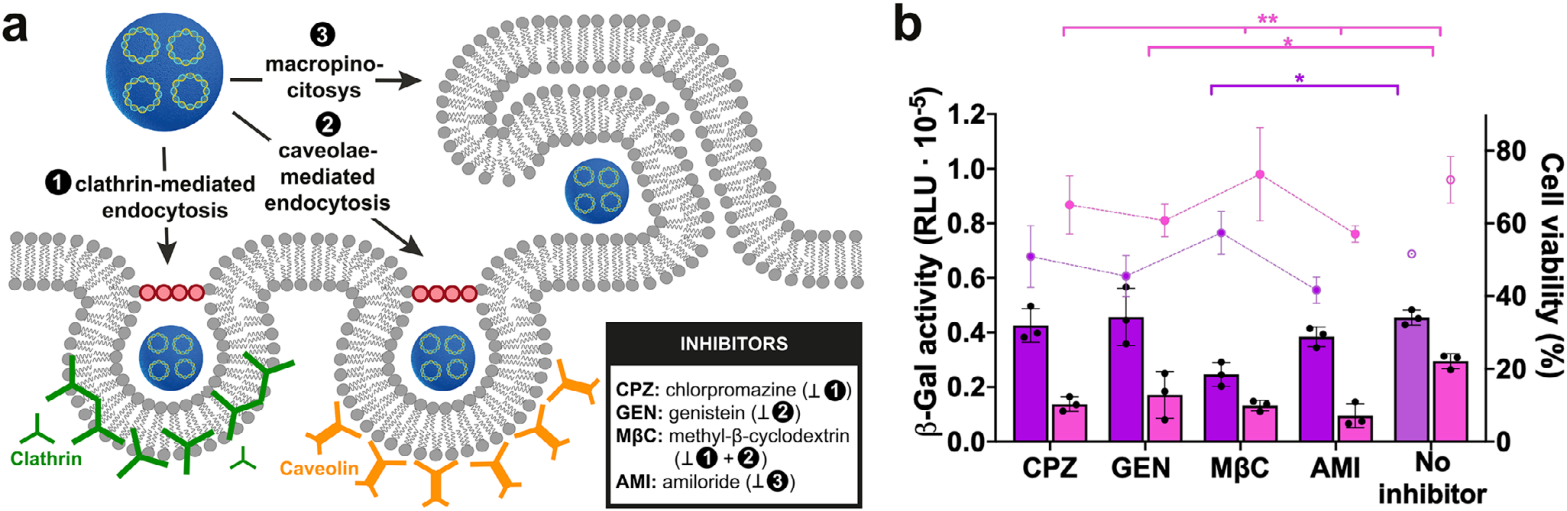
Internalization mechanisms of DNA‐loaded lipopeptide synthetic cells. (a) Schematic representation of the main endocytic pathways (clathrin‐mediated, caveolae‐mediated, and macropinocytosis) for the lipopeptide/pDNA complexes, along with their corresponding pharmacological inhibitors [chlorpromazine (CPZ) for clathrin‐mediated; genistein (GEN) for caveolae‐mediated; methyl‐β‐cyclodextrin (MβC), for both clathrin‐ and caveolae‐mediated; and amiloride (AMI) for macropinocytosis]. (b) β‐Gal activity and cell viability of HEK 293T cells pre‐treated or not (no inhibitor) with endocytosis inhibitors and subsequently transfected with lipopeptide (**LP1** (purple) or **LP2** (magenta)/pDNA (p*lac*Z) complexes. Values (*n* = 4) are presented as mean ± standard deviation (SD).

Given that the internalization pathway is a crucial determinant of transfection success [39, 40], it becomes clear that this step governs the efficiency of cellular uptake and subsequent intracellular trafficking, ultimately shaping the overall outcome of gene delivery [40]. To further elucidate the internalization mechanisms involved in the cellular uptake of the lipopeptide vesicular systems, β‐gal expression was quantified after p*lac*Z transfection at the optimal concentration for each lipopeptide (40 µg/mL for **LP1**; 20 µg/mL for **LP2**), following treatment with various endocytosis inhibitors (Figure 4) [40, 41]. For **LP1**, a significant decrease in β‐gal activity was observed in cells pretreated with methyl‐β‐cyclodextrin (MβC; 24646.417 ± 4409.830) – an inhibitor for both clathrin‐ and caveolae‐mediated cellular uptake – compared with untreated cells (45452.750 ± 2751.437). This suggests that uptake of **LP1**/p*lac*Z complexes occur predominantly through clathrin‐ and caveolae‐dependent endocytosis pathways. In contrast, all inhibitors tested for **LP2** produced a significant reduction in β‐Gal activity relative to the untreated control (29486.417 ± 2754.047), indicating that **LP2**/p*lac*Z complexes may be internalized through several endocytic routes rather than a single dominant pathway. It is worth noting that the cytotoxic effects of the inhibitors were evaluated at two time points – immediately after incubation and 24 h post‐incubation (corresponding to the transfection time) – using the CCK8 assay (Figure S11). Cell viability was higher at the latter time point.

##### 2.4.2.4 Transfection Efficiency of Lipopeptide/pGFP Complexes

**FIGURE 5:**
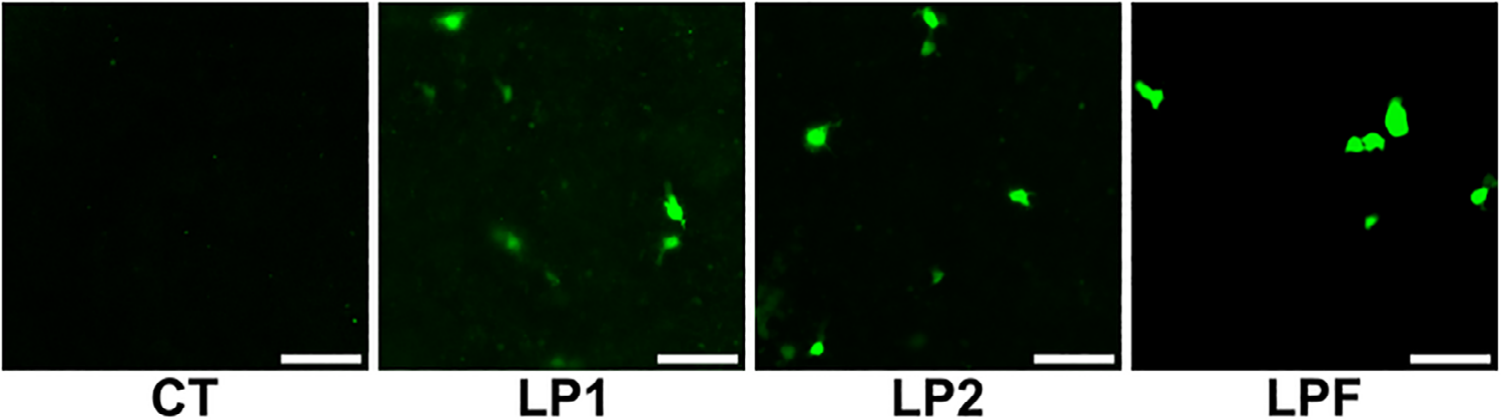
Fluorescence microscopy images (magnification: 20X; scale bar: 100 µm) showing GFP expression after transfection of HEK 293T with pGFP lipoplex complexes of **LP1** (40 µg/mL) or **LP2** (30 µg/mL). Untrasfected and LPF‐transfected cells were used as negative (CT) and positive (LPF) controls, respectively.

We next evaluated the versatility of our lipopeptide carriers by examining their ability to deliver a different nucleic acid (Figure 5). Plasmid length is a key factor that directly influences transfection efficiency [42]. In general, smaller plasmids – such as pGFP (5.8 kb) – achieve higher transfection efficiency than larger ones (e.g., p*lac*;Z; 7.8 kb), as their reduced size facilitates DNA condensation and packaging into smaller, more stable lipoplex complexes [43]. Moreover, shorter plasmids can diffuse more rapidly within the cytoplasm and undergo more efficient cellular internalization. Considering these factors, we investigated the cellular uptake of pGFP mediated by the lipopeptide (**LP1** and **LP2**) vesicular structures. The presence of GFP fluorescence within cells confirmed successful transfection of the pGFP plasmid (Figure 5, Figure S12). These results not only highlight the intrinsic activity of **LP1** and **LP2** but also validate that the optimal conditions established during the initial screening with p*lac*Z are robust and highly effective for a different reporter gene.

#### 2.4.3 Mesenchymal Stem Cells (MSCs)

Building on the robust results obtained in HEK 293T cells, we subsequently extended our strategy to more complex and clinically relevant cellular models, including mesenchymal stem cells (MSCs). MSCs are widely regarded as ‘hard to transfect cells’ [44] – a category they share with immune and neuronal cell types – due to the inherent difficulty of delivering exogenous material into them. MSCs are non‐hematopoietic, fibroblast‐like, multipotent adult cells characterized by self‐renewal, low immunogenicity, and strong immunomodulatory and differentiation capacities. These features make them attractive candidates for cell‐based therapies aimed at tissue regeneration and immune modulation. However, their therapeutic potential is often limited by impaired proliferation and differentiation capacity associated with donor aging and prolonged in vitro expansion. Consequently, MSC‐based gene therapy has emerged as a promising approach to enhance their functional properties and overall therapeutic efficacy.

##### 2.4.3.1 Transfection Efficiency of Lipopeptide/placZ Complexes

**FIGURE 6:**
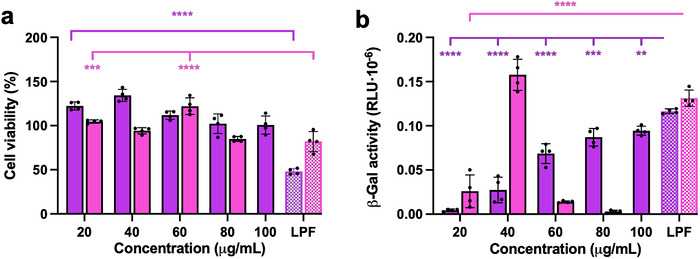
Transfection of p*lac*Z into iMSCs mediated by cationic lipopeptide‐based synthetic cells. (a) Cell viability of lipopeptide [**LP1** (*purple*) or **LP2** (*magenta*)]/pDNA (p*lac*Z) complexes. (b) β‐Gal activity of **LP1** (*purple*) and **LP2** (*magenta*) lipopeptide/p*lac*Z complexes formed at different concentrations. The commercial reagent Lipofectamine Stem (LPF) served as a positive transfection control. Values (*n* = 4) are presented as mean ± standard deviation (SD). * Depicts p < 0.05*, **p < 0.01, ***p < 0.001 and ****p < 0.0001 compared with the indicated LPF group.

In this context, the gene‐transfer efficiency of **LP1** and **LP2** lipopeptides was evaluated in immortalized MSCs (iMSCs) [45] – cells engineered to bypass replicative senescence and enable sustained in vitro expansion – using the same reporter plasmids encoding β‐Gal (p*lac*Z) and GFP (pGFP) as previously described (Figure 6). For p*lac*Z, initial experiments with **LP1** showed that the highest transfection efficiency was achieved at the highest concentration tested (100 µg/mL). However, this condition (94401.250 ± 5056.340) did not reach the levels obtained with the Lipofectamine Stem (LPF) positive control (116483.000 ± 2559.830). Notably, all tested concentrations resulted in cell viabilities above 75% and were significantly higher than the positive control (48.080 ± 3.315% (p < 0.0001). **LP2** cytotoxicity and transfection efficiency were evaluated across the same concentration range. In this case, the highest transfection efficiency (157722.250 ± 17 560.520) was observed at an intermediate concentration (40 µg/mL), significantly exceeding that of the positive control (131238.000 ± 9223.900, p<0.05). All tested concentrations maintained cell viabilities above 75%, compared to the control (82.061 ± 11.435%).

##### 2.4.3.2 Cytochemical Detection of β‐Galactosidase Activity

To complement the p*lac*Z reporter gene analysis, a histomorphometric evaluation was performed using a β‐Gal staining kit. In situ transfection was visualized by inverted microscopy, and transfection efficiency was qualitatively assessed based on staining intensity (Figure S13).

##### 2.4.3.3 Cytotoxicity and Transfection Efficiency of Lipopeptide/pGFP Complexes

**FIGURE 7:**
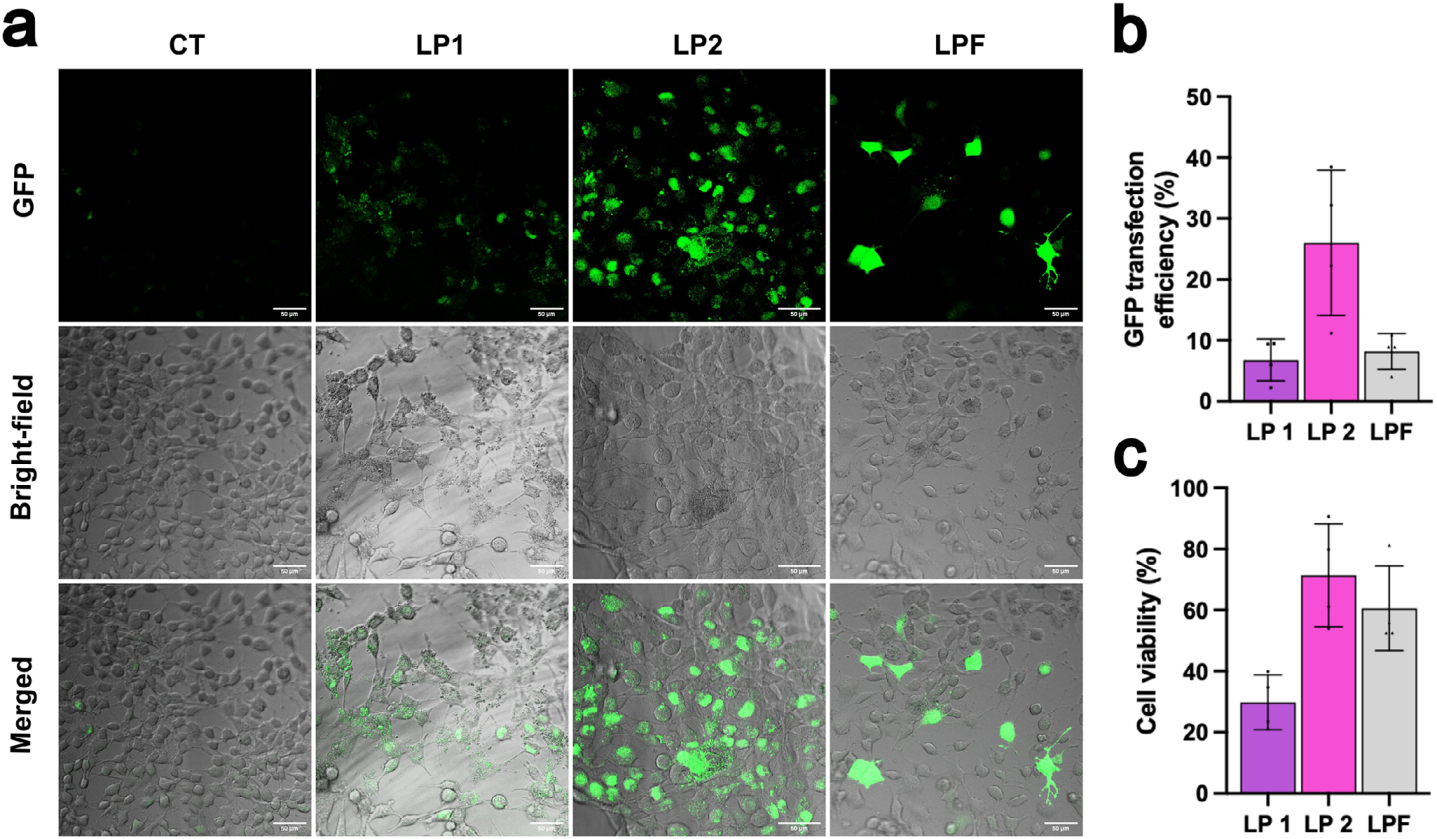
Cytotoxicity and transfection efficiency of lipopeptide/pGFP complexes in MSCs. (a) Representative fluorescence microscopy images (magnification: 20X; scale bar: 50 µm) showing GFP expression (*green*) after transfection of iMSCs with **LP1** and **LP2** lipoplexes at 100 and 40 µg/mL, respectively. Cells cultured in DMEM (CT) and cells transfected with LPF (LPF) were used as negative and positive control, respectively. Quantification of (b) GFP transfection efficiency and (c) cell viability of lipopeptide [**LP1** (*purple*) or **LP2** (*magenta*)]/pGFP complexes. The commercial reagent Lipofectamine Stem (LPF) served as a positive transfection control. Values (*n* = 4) are presented as mean ± standard deviation (SD). * Depicts p<0.05 when compared with denoted LPF group.

In parallel, in situ transfection of iMSCs was evaluated by confocal microscopy (Nikon, Tokyo, Japan) based on GFP expression (Figure 7a). Transfection efficiency was quantified using ImageJ software by calculating the percentage of GFP‐positive cells relative to the total number of cells in four randomly selected fields per condition (Figure 7b). Cell viability was determined by comparing the number of cells in each sample with that of the untransfected control (Figure 7c). In this analysis, **LP1**/pGFP complexes (6.773 ± 3.439%) achieved levels of GFP positivity comparable to those obtained with the positive control LPF (8.207 ± 2.929%), although they exhibited higher cytotoxicity than LPF. In contrast, **LP2** showed a 3.2‐fold‐increase in GFP expression relative to the positive control (26.023 ± 11.930%) (*p* < 0.05). Moreover, cell viability in the presence of **LP2**/pGFP complexes was higher than in the LPF group (*p* = 0.0217).

## 3 Conclusions

In summary, we have developed a new class of functional lipopeptides that self‐assemble into micron‐sized vesicles, which both mimic cellular compartments and act as potent non‐viral vectors. These membrane‐bounded assemblies efficiently sequester and deliver nucleic acids, achieving superior transfection efficiencies with minimal cytotoxicity in HEK 293T cells, while also demonstrating strong performance in more challenging models such as MSCs. We envision that these self‐assembling lipopeptides could enable the creation of robust, biocompatible, and programmable synthetic cells capable of delivering diverse nucleic acids into hard‐to‐transfect cells, thereby expanding the versatility and applications of traditional non‐viral vectors.

## 4 Experimental Section

### 4.1 General Methods

All reagents commercially supplied were used without further purification. 2‐Chlorotritylchloride resin (100‐200 mesh) was purchased from Fluorochem. Poly‐Prep chromatography columns were obtained from Bio‐Rad. Fmoc‐L‐Lys(Boc)‐OH, Fmoc‐L‐Lys(Fmoc)‐OH, 1,1,1,3,3,3‐Hexafluoro‐2‐propanol (HFIP) and sodium 8‐hydroxypirene‐1,3,6‐trisulfonate (HPTS) were purchased from BLD Pharmatech. Fmoc‐L‐Dap(Fmoc)‐OH was obtained from Angene. HOBt was purchased from Merck. HBTU was acquired from Manchester Organics. DIPEA was purchased from TCI Chemicals. Laurdan, Lipofectamine, and Lipofectamine Stem were obtained from Invitrogen. Dichloromethane (DCM) and methyl‐β‐ciclodextrin (MβC) were purchased from Merck. Dimethylformamide (DMF), 4‐methylpiperidine (4‐MP), chlorpromazine hydrochloride (CPZ), and genistein (GEN) were acquired form Fischer. Amiloride (AMI) was purchased from MedChemExpress. HEK 293T cells were kindly donated by Dr. Juan Antonio Fafián Labora (CICA, UDC). iMSCs were kindly donated by Prof. Silvia Díaz Prado (CICA, UDC). Plasmid pCMV‐SPORT‐βgal (p*lac*Z; 7853 bp) was obtained from Gibco Thermo Fisher Scientific (Madrid, Spain). Plasmid pCMV6‐A‐GFP (pGFP; 5806 bp) was obtained from OriGene Technologies (MD, USA). HPLC analysis was carried out on an Agilent 1260 Infinity II LC system (Agilent Technologies, USA) using an Eclipse Plus C8 analytical column with Phase A/Phase B gradients [Phase A: H_2_O with 0.1% TFA; Phase B: ACN with 0.1% TFA]. HPLC purification was carried out on an Agilent 1260 Infinity II LC system (Agilent Technologies, USA) using a Zorbax SBC18 semipreparative column with Phase A/Phase B gradients [Phase A: H_2_O with 0.1% TFA; Phase B: ACN with 0.1% TFA]. Electrospray ionization mass spectra (ESI‐Orbitrap‐MS) were obtained on a MAT95XP instrument. Absorbance was measured using a Genesys 50 UV‐Vis spectrophotometer (Thermo Scientific, Spain). Dynamic Light Scattering (DLS) and Electrophoretic Light Scattering (ELS) were measured using a Malvern Zeta‐Sizer (Malvern Instruments, UK). Microfluidic experiments were performed using the TAMARA platform (Inside Therapeutics, France). Phase‐contrast microscopy images were obtained from an Olympus microscope model BX53, using a 100x oil immersion objective and a phase‐contrast condenser (PH3). Images were processed using Fiji. Transmission electron microscopy (TEM) images were recorded on a JEOL JEM‐1010 100 KV microscope equipped with a tungsten thermoionic electron gun, using the standard copper grids developed by Electron Microscopy Sciences. Transfection images were obtained using either a confocal microscope A1R (Nikon) coupled to an inverted microscope model Eclipse Ti‐E (Nikon) or a Lionheart FX Digital Microscope. HPLC spectra were recorded using an Agilent 1260 Infinity II. Plates were analysed using a Synergy HTX plate reader (Biotek, Winooski, USA). CD spectra were obtained using a JASCO J‐1500 spectrophotometer, in a Quartz 400 µL cuvette with path length of 2 cm. Infrared (IR) spectra were recorded on a Bruker Vector 22 FTIR spectrometer.

### 4.2 Synthesis of Lipopeptides Dioleoyl‐DapKKK (**LP1**) and Dioleoyl‐KKKK (**LP2**)

#### 4.2.1 Attachment of the First Amino Acid to the Resin

Pre‐swollen 2‐chlorotrityl chloride (2‐CTC) resin (200.5 mg, 300.8 µmol) located in a Poly‐Prep column was treated with a solution of Fmoc‐L‐Lys(Boc)‐OH (169.1 mg, 360.9 µmol) in 2 mL of DCM containing DIPEA (246.8 µL). The suspension was gently shaken for 45 min at rt and washed with DCM (2 × 3 mL × 2 min).

#### 4.2.2 Resin Capping

Residual chlorides were capped by adding 5 mL of a DCM/MeOH/DIPEA mixture (4.25/0.5/0.25) and shaking for 20 min at rt. The resin was subsequently washed with DCM (2 × 5 mL × 2 min).

#### 4.2.3 Determination of Resin Loading

Resin loading was determined following standard Fmoc test protocols. In a typical experiment, 1.9 mg of amino acid‐resin were weighed in an Eppendorf tube. Next, 1 mL of a 20% solution of 4‐MP in DMF was added and left agitating for 30 min. Solution was diluted 1:10 and the absorbance was measured at 301 nm versus a DMF blank. Loading was calculated using the formula:

$$\frac{(Abs_{sample}-Abs_{ref})\times 16.4}{mg\;resin}$$

affording a final loading of 0.78 mmol/g. This loading value was considered for further couplings.

#### 4.2.4 Peptide Elongation

Fmoc deprotection was carried out by treating the capped resin with a solution of 20% of 4‐MP in DMF (3 mL) for 30 min at rt, followed by washing with DMF (2 × 3 mL × 2 min) and DCM (2 × 3 mL × 2 min).

Second amino acid attachment was next performed by treating the previous Fmoc unprotected resin with a preactivated solution (premixed for 1 min prior to addition) of Fmoc‐Lys(Fmoc)‐OH (366.0 mg, 781.2 µmol), HBTU (296.3 mg, 781.2), HOBt (119.6 mg, 781.2 µmol) and DIPEA (267.2 µL, 1562.5 µmol) in DMF (3 mL). The mixture was then agitated for 45 min at rt. Afterwards, the resin was washed with DMF (2 × 3 mL × 2 min) and DCM (2 × 3 mL × 2 min).

Fmoc‐deprotection and third amino acid attachment were performed with identical conditions to the ones described above, finally affording the tripeptide Fmoc‐Lys(Boc)‐Lys(Boc)‐Lys(Boc)‐ linked to the resin.

After attachment of the first three amino acids, the resin was split into two equal portions to further generate **LP1** and **LP2** by Fmoc deprotection with 20% of 4‐MP in DMF (3 mL) and subsequent coupling with the corresponding Fmoc‐protected amino acid.

For **LP1**, 100.0 mg of preloaded resin were taken and treated with a preactivated solution (premixed for 1 min prior to addition) of Fmoc‐Dap(Fmoc)‐OH (171.2 mg, 312.0), HBTU (118.3 mg, 312.0 µmol), HOBt (47.8 mg, 312.0 µmol) and DIPEA (106.7 µL, 624.0 µmol) in DMF (3 mL). The mixture was then agitated for 45 min at rt. Afterwards, the resin was washed with DMF (2 × 3 mL × 2 min) and DCM (2 × 3 mL × 2 min).

For **LP2**, 100.0 mg of preloaded resin were taken and treated with a preactivated solution (premixed for 1 min prior to addition) of Fmoc‐Lys(Fmoc)‐OH (184.3 mg, 312.0 µmol), HBTU (118.3 mg, 312.0 µmol), HOBt (47.8 mg, 312.0 µmol) and DIPEA (106.7 µL, 624.0 µmol) in DMF (3 mL). The mixture was then agitated for 45 min at rt. Afterwards, the resin was washed with DMF (2 × 3 mL × 2 min) and DCM (2 × 3 mL × 2 min).

#### 4.2.5 Coupling of Fatty Acid Chains

For each lipopeptide, 39.0 mg of previously loaded resin were taken (30.4 µmol). After Fmoc deprotection of the final amino acid – both in its N‐terminus and N‐lateral side with 20% of 4‐MP in DMF (2 mL) –, resin was treated with a solution of oleic acid (68.7 mg, 243.4 µmol), HATU (50.9 mg, 133.9 µmol) and DIPEA (121.7 µL, 486.7 µmol) in DCM (2 mL) and shaked for 2 h at rt.

#### 4.2.6 Cleavage of the Lipopeptides From the Resin

Lipopeptides were next cleaved from the resin by treatment with a solution of 20% of HFIP in DCM (2 mL) for 2 h. Afterwards, the resin was washed with DCM (2 × 1 mL), and the combined filtrates were concentrated under reduced pressure, obtaining a pale yellowish oil for each lipopeptide.

#### 4.2.7 Release of Protecting Groups

Boc protecting groups were then removed by dissolving the previous residue in DCM (3 mL) at 0 °C and adding TFA dropwise until a final concentration of 10% TFA in DCM was achieved. The mixture was stirred at rt for 30 min, and then concentrated under reduced pressure to yield each crude lipopeptide as a yellow oil.

### 4.3 Purification of Lipopeptides Dioleoyl‐DapKKK (**LP1**) and Dioleoyl‐KKKK (**LP2**)

Crude lipopeptides (**LP1** or **LP2**) were dissolved in MeOH and purified by HPLC (50–85% Phase B in Phase A, 0–5 min; 85–95% Phase B in Phase A, 5–15 min; 95–100% Phase B in Phase A, 15–20 min; 100–50% Phase B in Phase A, 20–25 min).

For **LP1**, 25.1 mg were afforded as a colorless oil [81%; t_R_ = 4.75 min (Figure S1), Eclipse Plus C8 analytical column (50–95% Phase B in Phase A, 0–1 min; 95% Phase B in Phase A, 1–8 min; 95–100% Phase B in Phase A, 8–10 min; 100% Phase B in Phase A, 10–13 min; 100–50% Phase B in Phase A, 13–15 min)]. MS (ESI) calculated for (**LP1**) C_57_H_108_N_8_O_7_ [M+H]^+^: 1017.83, found 1017.84 (Figure S2). The FTIR spectrum (Figure S4) shows the characteristic amide bands, including amide I (C═O stretching, ∼1650 cm^−^^1^), amide II (N─H bending/C─N stretching, ∼1540 cm^−^^1^), and amide A (N─H stretching, ∼3300 cm^−^^1^), together with aliphatic C–H stretching bands (∼2950–2850 cm^−^^1^), consistent with the lipopeptide structure. The CD spectrum (Figure S5) displays a strong negative peak in the far‐UV region (around 200 nm) and very low ellipticity (near zero) at longer wavelengths (above 210 nm), indicative of a predominantly disordered or “random coil” conformation.

For **LP2**, 25.0 mg were afforded as a colorless oil [78%; t_R_ = 4.95 min (Figure S1), Eclipse Plus C8 analytical column. (50–95% Phase B in Phase A, 0–1 min; 95% Phase B in Phase A, 1–8 min; 95–100% Phase B in Phase A, 8–10 min; 100% Phase B in Phase A, 10–13 min; 100–50% Phase B in Phase A, 13–15 min)]. MS (ESI) calculated for (**LP2**) C_60_H_114_N_8_O_7_ [M+H]^+^: 1059.88, found 1059.88 (Figure S3). The FTIR spectrum (Figure S4) shows the characteristic amide bands, including amide I (C = O stretching, ∼1650 cm^−^^1^), amide II (N–H bending/C–N stretching, ∼1540 cm^−^^1^), and amide A (N–H stretching, ∼3300 cm^−^^1^), together with aliphatic C–H stretching bands (∼2950–2850 cm^−^^1^), consistent with the lipopeptide structure. The CD spectrum (Figure S5) displays a strong negative peak in the far‐UV region (around 200 nm) and very low ellipticity (near zero) at longer wavelengths (above 210 nm), indicative of a predominantly disordered or “random coil” conformation.

### 4.4 Characterization of Lipopeptide Vesicles Constituted by Dioleoyl‐DapKKK (**LP1**) and Dioleoyl‐KKKK (**LP2**)

#### 4.4.1 Vesicle Formation

##### 4.4.1.1 Hydration Method

10 µL of a 50 mM solution of the lipopeptide (**LP1** or **LP2**) in a mixture 1:1 MeOH:CHCl_3_ were added to a 1 mL glass vial and dried under N_2_ flow to form a lipid film. The corresponding film was then hydrated using 100 µL of a 100 mM PBS (pH 7.4) solution for 1 h at rt. Afterwards, the sample was analyzed by phase‐contrast microscopy, observing the formation of the expected vesicles (Figure 2a, Figure S6).

##### 4.4.1.2 Microfluidics

Vesicle nanoparticles were generated using TAMARA, a microfluidic‐based nanoparticle formulation system. For that, a solution of the lipopeptide (**LP1** or **LP2**) in EtOH (final concentration: 10 mg/mL) was combined with a 1 mM PBS (pH 7.4) solution in a ratio of 1:3 and a total flow rate of 4 mL/min to obtain the corresponding lipopeptide (**LP1** or **LP2**) nanoparticles (Final concentration: 2.5 mg/mL). The size of the vesicles was characterized by dynamic light scattering (DLS), while the surface charge was determined by zeta potential analysis (Figure 2e).

#### 4.4.2 Encapsulation Study

##### 4.4.2.1 Inverse Emulsion

60 µL of a 20 mM solution of the lipopeptide (**LP1** or **LP2**) in MeOH:CHCl_3_ were added to a 1 mL glass vial and dried under N_2_ flow to form a lipid film. Then, 200 µL of mineral oil were added and the mixture was sonicated for 1 h. After sonication, 100 µL of the lipopeptide‐oil mixture were added to a 1 mL Eppendorf tube containing 10 µL of an upper buffer solution (50 µM HPTS + 200 mM sucrose in 100 mM HEPES buffer pH 7.5). The mixture was flicked until a cloudy emulsion was observed. This emulsion was carefully layered onto 100 µL of a lower buffer solution (200 mM glucose in 100 mM HEPES buffer pH 7.5) in a separate 1 mL Eppendorf tube, ensuring that the lipopeptide‐oil solution floated on top. Sample was left to equilibrate for 10 min and then centrifuged at 10000 g for 10 min. Finally, the oil layer was aspirated, leaving the vesicle‐containing aqueous phase at the bottom. This lower phase contained the lipopeptide vesicles encapsulating the HPTS, which was analyzed by fluorescence microscopy (Figure 2c, Figure S8).

#### 4.4.3 Determination of the Critical Aggregation Concentrations (CACs)

The critical aggregation concentration (CAC) of the lipopeptides (**LP1** or **LP2**) was assessed using the solvatochromic fluorescent dye Laurdan, following a previously described protocol [30]. Serial dilutions of a lipopeptide stock solution in Milli‐Q water (1 mM) were prepared at concentrations ranging from 0 to 100 µM, with a final volume of 200 µL. The samples were then incubated for 1 h at rt. Subsequently, 2.5 µL of a 100 µM Laurdan solution in EtOH was added to each sample prior measuring the absorbance. Solutions were transferred to a quartz cuvette and measured using a spectrofluorometer. Samples were excited at 364 nm and emission spectrum was acquired ranging 420–500 nm. The GP was calculated following the next equation:

$$GP=\frac{I_{440}-I_{490}}{I_{440}+I_{490}}$$

I_440_ and I_490_ stand for the fluorescence intensities at both wavelengths. Values of GP were represented against the LP concentration for each dilution. The CACs of the lipopeptides **LP1** and **LP2** were determined to be 29.6 and 28.4 µM, respectively, by intersecting the two straight lines corresponding to monomer and vesicle regions (Figure 2d).

#### 4.4.4 Transmission Electron Microscopy (TEM) Measurements

3.5 µL of a 2.5 mg/mL solution of lipopeptide (**LP1** or **LP2**) vesicles in 100 mM PBS at pH 7.4 (previously synthesized by using the TAMARA microfluidic platform) was deposited on the surface of a copper grid (formvar/carbon‐coated, 400 mesh copper). The solution was let to sit for 10 s before being washed with 10 drops of filtered distilled H_2_O. Subsequently, staining was done with 3 drops of 1% w/w uranyl acetate. The stain was allowed to sit for 10 s before wicking away with filter paper. Samples were then imaged via TEM, revealing populations of spherical compartments that were between 50–60 nm for **LP1** and 60–70 nm for **LP2**, consistent with the vesicle architecture (Figure 2b, Figure S7).

#### 4.4.5 Dynamic Light Scattering (DLS) and Zeta Potential Measurements

Lipopeptide **(LP1** or **LP2)** vesicles [final concentration: 2.5 mg/mL in 1 mM PBS (pH 7.4)], previously obtained by either hydration or microfluidic formation, were analyzed to determine their size and charge characteristics. Before measurement, vesicles were diluted to a final concentration of 0.25 mg/mL using 1 mM PBS (pH 7.4). The sample was then transferred to a DTS0012 cuvette Malvern Instruments to obtain size distribution. Subsequently, the same sample was transferred to a DTS1070 Zeta potential cell to obtain zeta potential (Figure 2e).

### 4.5 Transfection Experiments

#### 4.5.1 Plasmid Isolation and Purification

Plasmids encoding for β‐galactosidase (p*lacZ*) or green fluorescent protein (pGFP) were propagated, purified, and quantified following standard procedures. All pDNA solutions were diluted with nuclease‐free water and stored at −20 °C until use.

#### 4.5.2 Lipopeptide/pDNA Complexes Formation

##### 4.5.2.1 Lipopeptide/placZ Complexes Formation: Complexation Assay

Lipopeptide (**LP1** or **LP2**) was dissolved in MeOH to create a lipid film with a desired concentration. After that, the organic solvent was removed under nitrogen flow, forming the lipid film. The film was hydrated with 1 mL of OptiMEM Reduced Serum Medium (1X). Various concentrations (20–200 µg/mL; Final volume: 50 µL) were prepared to carry out different analysis by dilution with nuclease‐free water (Ambion AM9937). Then, 1.05 µL of a p*lacZ* stock solution (710 µg/mL) were added, followed by incubation for 30 min at rt. Next, 3 µL of a 200X SYBR Gold stock solution were added to each sample, and then they were incubated for 10 min at rt in dark. Afterwards, 270 µL of a 10 mM HEPES buffer (pH 7.4) solution were added to each sample, and these were transferred to a 96‐well plate (100 µL per well). Fluorescence was measured using a Synergy HTX plate reader, using 485/20 nm excitation and 528/20 nm emission filters (Figure 2f, Figure S10).

##### 4.5.2.2 Lipopeptide/pDNA Complexes Formation for Cellular Uptake

Lipopeptide (**LP1** or **LP2**) was dissolved in MeOH in order to create a lipid film with a desired concentration. Afterwards, the organic solvent was removed under nitrogen flow, obtaining the corresponding lipid film. The film was then hydrated with 1 mL of OptiMEM Reduced Serum Medium (1X). Lipopeptide/pDNA complexes were then formed by mixing p*lacZ* or pGFP plasmid (742 ng) with the corresponding lipopeptide (**LP1** or **LP2**) solution to obtain various concentrations (2.5–50 µg/mL), thus being able to carry out different analysis. All mixtures were allowed to complex for 30 min at rt before being used in different experiments. Lipofectamine was used as positive control; their corresponding lipoplexes were prepared with the same plasmid concentrations and the recommended reagent volume, according to the manufacturer instructions.

#### 4.5.3 Cytotoxicity and Transfection Efficiency of Lipopeptide/pDNA Complexes

HEK 293T (75000 cells/well in 48 well‐plates) or iMSCs (10000 cells/well in 96 well‐plates) were allowed to attach for 24 h at 37 °C before the experiments. Then, cells were transfected with lipopeptide (**LP1** or **LP2**)/pDNA (p*lac*Z or pGFP) complexes and incubated for 24 h at 37 °C and 5% CO_2_. Untransfected cells (negative control) or cells transfected with Lipofectamine (positive control) were assessed in parallel. All conditions were evaluated by quadrupled.

##### 4.5.3.1 Cell Viability

Viability of the cell monolayers was monitored at 24 h using the CCK8 reagent, following the supplier's instructions (Neo Biotech, France). Briefly, cells were washed with 1X PBS (pH 7.4), and then incubated with CCK8 reagent (100 µL; 1:10 dilution in DMEM) for at least 2 h at 37 °C and 5% CO_2_. The absorbance (Abs) signal at 450 nm was measured in a Sinergy HTX Plate reader. Cell viability was obtained with the following formula:

$$Cell\;viability\;\;\left(\%\right)=\frac{Abs_{sample}\;450\;nm}{Abs_{control\;}450\;nm}\;\times 100$$

##### 4.5.3.2 Transfection Efficiency (pLacZ)

 β‐galactosidase activity achieved with p*lacZ* lipoplexes was evaluated at 24 h post‐transfection by using the β‐glo reagent (100 µL 1:1 dilution in DMEM) of β‐glo kit (Promega, Madison, WI, USA). Cells were incubated for 30 min at rt without light. Luminescence was measured by using the plate reader mentioned above (parameters: 135 gain). Endogenous β‐galactosidase activity of untransfected cells was used as a blank and subtracted from each condition with the following formula:

$$\begin{array}{c} \beta -Galactosidade\;activity\;\;\left(RLU\right)\\ =RLU\;\left(sample\right)-RLU\;\left(negative\;control\right) \end{array}$$

##### 4.5.3.3 Transfection Efficiency (pGFP)

After 24 h of transfection, cells were washed with 1X PBS (pH 7.4). GFP positive cells were imaged by using either a Lionheart FX Digital Microscope (Figure 5) or a confocal microscope A1R (Nikon) coupled to an inverted microscope model Eclipse Ti‐E (Nikon) (Figure 7 and Figure S12), using GFP filters: Em. 469/35 nm, Ex. 525/39 nm.

#### 4.5.4 Cytochemical Detection of β‐Galactosidase Activity

Cytochemical evaluation of β‐galactosidase activity was performed by using the β‐gal staining kit. At 24 h post‐transfection, the medium was removed, and the cells were washed with 1X PBS (pH 7.4). Then, the cells were fixed with 2.5% formaldehyde in 1X PBS (pH 7.4) (250 µL) and subsequently incubated for 15 min at rt. Afterwards, the cells were washed at least three times with 1X PBS (pH 7.4) and stained with the X‐gal reagent (250 µL), previously prepared following the instructions of the manufacturer (Roche, Switzerland). Cells were incubated for at least 3 h at 37 °C and 5% CO_2_. Finally, the transfection efficiency was analyzed with a Lionheart FX Digital Microscope (Figure 3C).

#### 4.5.5 Internalization Mechanism of Lipopeptide/pDNA Complexes

The internalization pathways of the lipopeptide/pDNA complexes were analyzed by measuring the β‐galactosidase activity in transfected HEK 293T cells after treatment with different endocytosis inhibitors. Initially, the cytotoxic effects of the inhibitors were evaluated at two time points – immediately after incubation and 24 h post‐incubation (corresponding to the transfection time) – using the CCK8 assay (Figure S11). Based on these results, HEK 293T cells (75000 cells/well) were seeded in 48‐well plates and incubated for 24 h prior to inhibitor treatment at optimized concentrations and exposure times: chlorpromazine (CPZ: clathrin‐mediated endocytosis inhibitor, 0.03 mM, 1 h), methyl‐β‐cyclodextrin (MβC: clathrin‐ and caveolae‐dependent endocytosis inhibitor, 2 mM, 10 min), genistein (GEN: caveolae‐mediated endocytosis inhibitor, 0.1 mM, 1 h), and amiloride (AMI: macropinocytosis inhibitor, 2 mM, 10 min) [11, 44].

Following inhibitor pretreatment, cells were transfected for 24 h with the lipopeptide (**LP1** or **LP2**)/pDNA complexes at concentrations of 40 and 30 µg/mL, respectively. Cells transfected in the absence of inhibitors and untransfected cells were included as control conditions. Each experimental condition was performed in triplicate (Figure 4). Cell viability and β‐galactosidase activity were quantified according to the protocols described in the section *Cytotoxicity and transfection efficiency of lipopeptide/pDNA complexes*.

## Author Contributions

F.A.S‐T., A.R‐B., A.R.‐R., and R.J.B conceived and designed the experiments. F.A.S‐T synthesized and characterized LP1 and LP2 and their self‐assembling properties. A.R‐B. carried out the in vitro biological assays. A.R.‐R. and R.J.B. secured funding. F.A.S‐T, A.R‐B., P.F.‐T., A.R.‐R., and R.J.B. analyzed the data and wrote the paper, contributing to the final version of the manuscript.

## Funding

This work was supported by Agencia Estatal de Investigación (PID2021‐128113NA‐I100, PID2021‐128461OB‐I00, CNS2024‐154660, and RYC2020‐030065‐I), and the Consellería de Cultura, Educación e Universidade da Xunta de Galicia (ED431F 2024/07 and ED431B 2023/60). The authors acknowledge funding for open‐access charges from Universidade da Coruña/Consorcio Interuniversitario de Galicia (CISUG).

## Conflicts of Interest

The authors declare no conflicts of interest.

## Supporting information




**Supporting File**: smll74067‐sup‐0001‐SuppMat.docx.

## Data Availability

The data that support the findings of this study are available from the corresponding author upon reasonable request.
