# Supplementary material for: Self‐Assembling Cationic Lipopeptides for the Construction of Functional Vesicular Gene‐Delivery Systems
Source: Small. 2026 Jun 5;22(42):e74067. doi: 10.1002/smll.74067 (PMC13410438; doi:10.1002/smll.74067)
Supplement: Supplementary file 1 — Supporting File: smll74067‐sup‐0001‐SuppMat.docx. [file SMLL-22-e74067-s001.docx]

Supporting Information

Self-Assembling Cationic Lipopeptides for the Construction of Functional Vesicular Gene-Delivery Systems

Federica A. Souto-Trinei,^[a],†^ Alba Ramil-Bouzas,^[a],[b],†^ Paco Fernández-Trillo,^[a]^ Ana Rey-Rico*^[b]^ and Roberto J. Brea*^[a]^

[a] F. A. Souto-Trinei, A. Ramil-Bouzas, Prof. P. Fernández-Trillo, Prof. R. J. Brea
Bioinspired Nanochemistry (BioNanoChem) Group
CICA - Centro Interdisciplinar de Química e Bioloxía
Departamento de Química, Facultad de Ciencias
Universidade da Coruña
Rúa As Carballeiras s/n, Campus de Elviña, 15071, A Coruña, Spain
E-mail: [roberto.brea@udc.es](mailto:roberto.brea@udc.es)
Homepage: <https://bionanochemlab.com>

[b] A. Ramil-Bouzas, Prof. A. Rey-Rico
Gene and Cell Therapy (G-Cel) Research Group
CICA - Centro Interdisciplinar de Química e Bioloxía
Departamento de Biología, Facultad de Ciencias
Universidade da Coruña
Rúa As Carballeiras s/n, Campus de Elviña, 15071, A Coruña, Spain
E-mail: [ana.rey.rico@udc.es](mailto:ana.rey.rico@udc.es)

[^†^] These authors contributed equally to this work

**Supplementary Schemes**

**Scheme 1.** Solid-phase peptide synthesis to obtain **LP1** and **LP2**.

**Supplementary Figures**

**Figure S1.** HPLC (210 nm) traces corresponding to lipopeptides **LP1** and **LP2**. Retention times (t_R_) were verified by mass spectrometry.


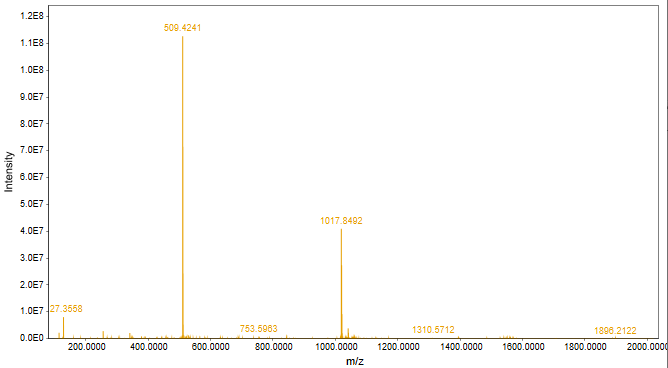


**Figure S2.** Mass spectrum of **LP1**.


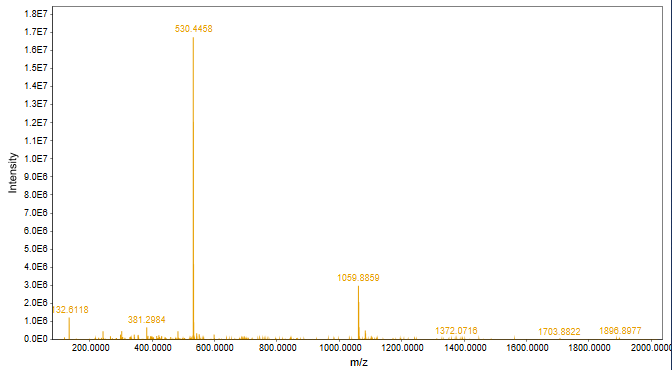


**Figure S3.** Mass spectrum of **LP2**.

**Figure S4.** FTIR spectra of **LP1** (*purple*) and **LP2** (*magenta*).

**Figure S5**. CD spectra of **LP1** (*purple*) and **LP2** (*magenta*) were recorded at 0.38 mM in Milli-Q water at rt.


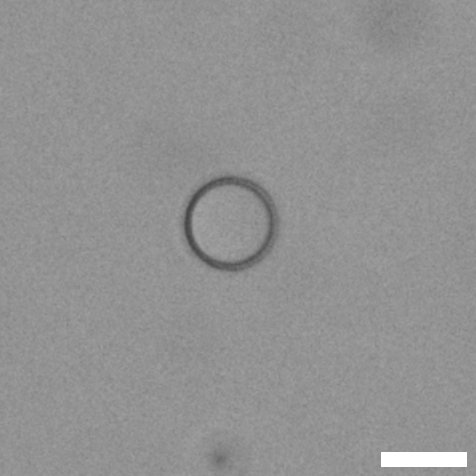


**Figure S6.** Phase-contrast microscopy image of **LP2** vesicles obtained from hydration in 100 mM PBS at pH 7.4. Scale bar denotes 5 µm.


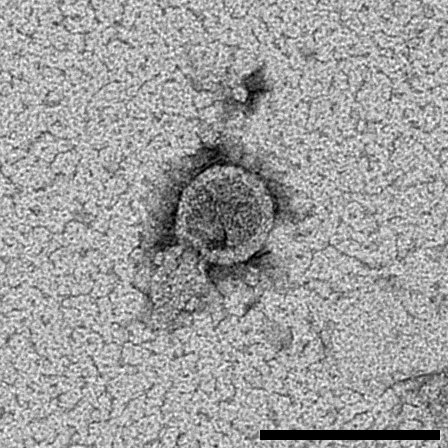


**Figure S7.** Transmission electron microscopy (TEM) image of negatively stained vesicles of **LP2**. Scale bar denotes 100 nm.


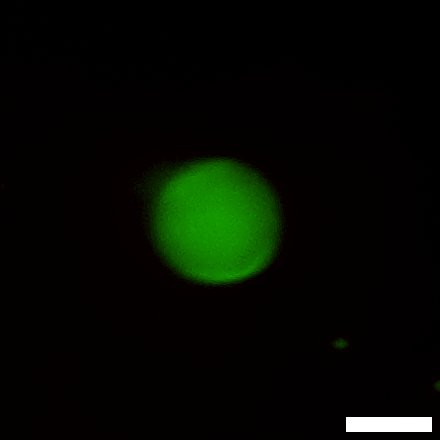


**Figure S8.** Fluorescence microscopy images demonstrating the encapsulation of HPTS in **LP2** vesicles. Scale bar denotes 5 µm.

**Figure S9.** Stability analysis of **LP1** (*purple*) and **LP2** (*magenta*) in FBS at different time points. HPLC spectra were recorded at 210 nm.

**Figure S10.** p*lacZ* complexation efficiency of lipopeptides **LP1** and **LP2**. * Depicts p<0.05, ** p<0.01, *** p<0.001, and **** p<0.0001, when compared with denoted plasmid control group.


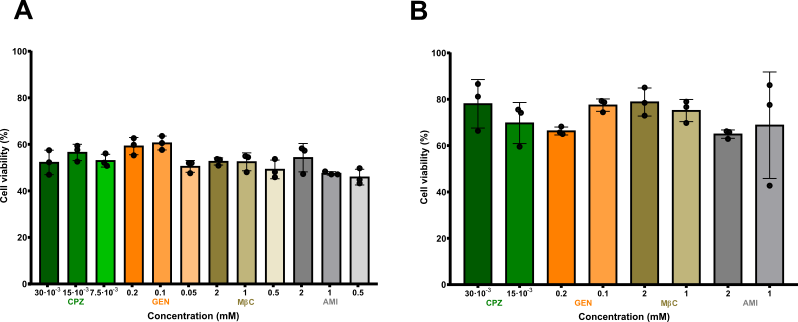


**Figure S11**. Cell viability of HEK 293T cells pretreated with endocytosis inhibitors at two time points: (A) immediately after incubation, and (B) 24 h post-incubation [CPZ: chlorpromazine; GEN: genistein; MβC: methyl-β-cyclodextrin; AMI: amiloride].


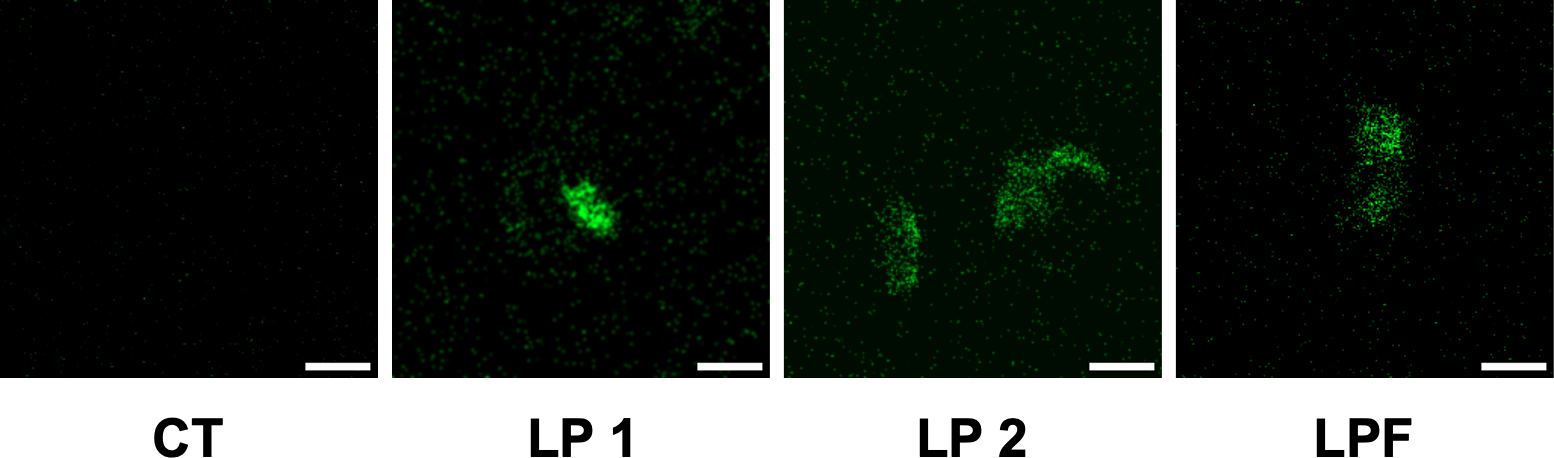


**Figure S12**. Fluorescence microscopy representative images (magnification 20X; scale bar: 100 μm) showing GFP expression after transfection of HEK 293T with **LP1** (40 μg/mL) and **LP2** (30 μg/mL). Cells transfected with LPF (LPF) and untransfected cells (CT) were used as positive and negative control, respectively.

**Figure S13**. Microscopy images of β-Gal staining (magnification: 20X; scale bar: 200 μm) showing successful p*lacZ* transfection in iMSCs after 3 h of incubation for both **LP1** and **LP2** / pDNA complexes. Cells transfected with LPF were used as positive control.
